# Recent 5-year Findings and Technological Advances in the Proteomic Study of HIV-associated Disorders

**DOI:** 10.1016/j.gpb.2016.11.002

**Published:** 2017-04-06

**Authors:** Lijun Zhang, Xiaofang Jia, Jun-O Jin, Hongzhou Lu, Zhimi Tan

**Affiliations:** Shanghai Public Health Clinical Center, Fudan University, Shanghai 201508, China

**Keywords:** HIV-1, Proteomics, Interaction, iTRAQ, SILAC

## Abstract

Human immunodeficiency virus-1 (**HIV-1**) mainly relies on host factors to complete its life cycle. Hence, it is very important to identify HIV-regulated host proteins. **Proteomics** is an excellent technique for this purpose because of its high throughput and sensitivity. In this review, we summarized current technological advances in proteomics, including general isobaric tags for relative and absolute quantitation (**iTRAQ**) and stable isotope labeling by amino acids in cell culture (**SILAC**), as well as subcellular proteomics and investigation of posttranslational modifications. Furthermore, we reviewed the applications of proteomics in the discovery of HIV-related diseases and HIV infection mechanisms. Proteins identified by proteomic studies might offer new avenues for the diagnosis and treatment of HIV infection and the related diseases.

## Introduction

Human immunodeficiency virus (HIV)/acquired immune deficiency syndrome (AIDS) remains one of the major infectious diseases affecting global health [1]. Great efforts and progresses have been made in the last decade. In particular, the application of antiretroviral therapy renders the HIV infection a chronic disease rather than a fatal one [2]. To inhibit HIV replication, almost all antiretroviral drugs target viral proteins, including reverse transcriptases, proteases, and integrases [3]. Although anti-HIV drugs are successful, the undesirable side effects and high cost are still big obstacles to the broad applications in AIDS patients [4]. Moreover, the virus quickly adapts and becomes resistant to these drugs [5], making it almost impossible to completely eradicate the virus because of HIV reservoirs [6]. Therefore, it is imperative to develop novel approaches and new therapeutic targets.

HIV-1 encodes only nine viral proteins (18 mature proteins) and thus it mainly depends on host cellular machinery to complete its life cycle [7]. So far, various host proteins have been found to play key roles during the HIV-1 life cycle [8], [9]. For example, HIV can enter target cells by interacting with cluster of differentiation-4 (CD4) [10] and coreceptors C–C chemokine receptor type 4 (CCR4) or CCR5 [11], as well as Golgi transport proteins [12]. Moreover, lens epithelium-derived growth factor (LEDGF) [13] and karyopherin [12] have been defined as HIV-1 integration proteins, whereas cyclin-dependent kinase 9 (CDK9)/cyclin T1 [14] and mediator complex 28 [12] contribute to HIV transcription. Furthermore, tumor susceptibility gene 101 [15] participates in HIV-1 budding. Therefore, these host proteins would be the targets for anti-HIV drugs [16]. For example, the CCR5 coreceptor is targeted by maraviroc, one type of drugs preventing the entry of HIV [17].

Although some targets have been identified by protein or genetic methods, our knowledge of HIV infection is still very limited because of the complexity of the HIV−host interactions and the host responses to infection. To discover cellular factors related to HIV-1 infection, high-throughput approaches, including genomic technologies [8], [17], [18], and proteomic approaches [19], [20], [21], [22], [23], [24], [25], [26], [27], [28], [29], [30], have been used. Given proteins are the ultimate effectors of most cellular functions proteomic approach is deemed to be one of the best ways to identify cellular factors related to HIV-1 infection and has already been widely used [31], [32]. Furthermore, proteomic technology can specially analyze protein posttranslational modifications (PTMs) occurring after viral infection [22], [33], [34], [35], [36]. In the current review, we summarized recent technological advances in proteomics and new discoveries implicating biomarkers of HIV-induced diseases and molecular mechanisms involved in HIV infection over the last 5 years.

## Technological advances

**Figure 1:**
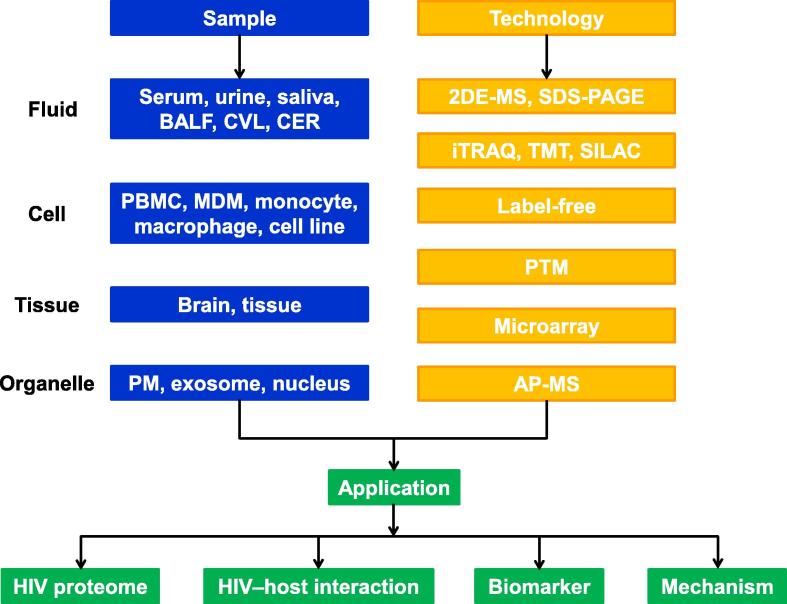
**Summary of current proteomic technologies and their applications in the study of HIV infection** A lot of proteomic technologies including gel-based, label or label-free proteomics are available for discovering diagnosis biomarkers and analyzing molecular mechanisms underlying HIV infection using samples such as body fluid, cells, and tissues. BALF, bronchoalveolar lavage fluid; CVL, cervicovaginal lavage; CER, Weck-Cel cervical sponge; PBMC, peripheral blood mononuclear cells; MDM, monocyte-derived macrophages; PM, plasma membrane; 2DE-MS, 2-dimensional electrophoresis mass spectrometry; SDS-PAGE, sodium dodecyl sulfate–polyacrylamide gel electrophoresis; iTRAQ, isobaric tags for relative and absolute quantitation; TMT, isobaric mass tagging; SILAC, stable isotope labeling by amino acids in cell culture; PTM, posttranslational modification; AP-MS, affinity purification-mass spectrometry; HIV, human immunodeficiency virus.

In the recent years, many proteomic technologies have been applied to investigate HIV infection (Figure 1). These include 2-dimensional electrophoresis (2DE), stable isotope labeling such as isobaric tags for relative and absolute quantitation (iTRAQ) or stable isotope labeling by amino acids in cell culture (SILAC), and label-free technologies (Table 1). In order to detect less abundant proteins, subcellular organelle separation can be performed before general proteomic studies. In addition, affinity purification−mass spectrometry (AP−MS) has been widely used to determine which proteins are involved in HIV−host interaction [19], [20], [21]. Furthermore, newly-developed technologies for PTM studies [22], [23] have also been used in the study of HIV infection.

### Subcellular proteomics

**Figure 2:**
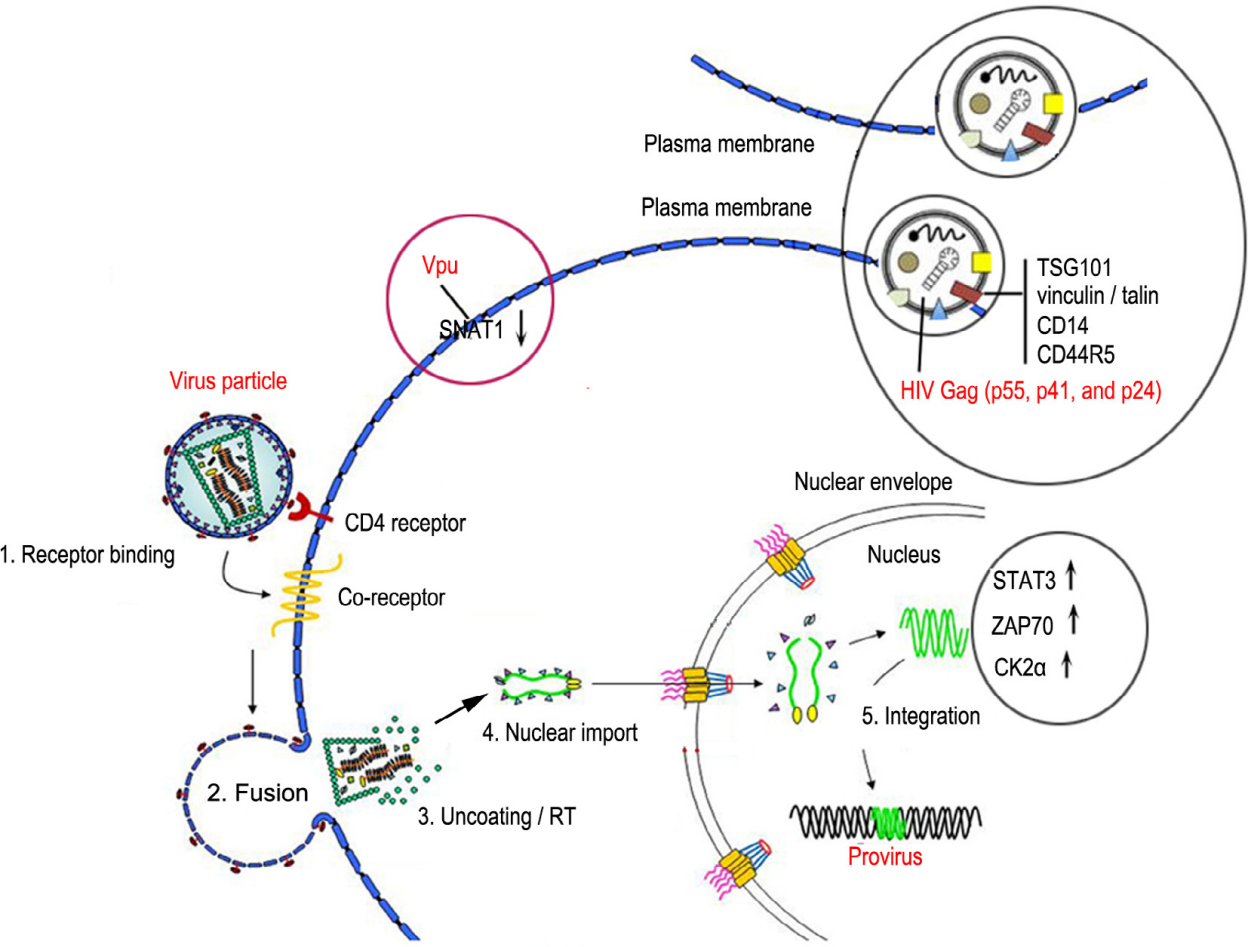
**Summary of the identified proteins involved in HIV life cycle and subcellular localizations up- and down-regulations reviewed in this work** Many host proteins get involved during HIV life cycle. Host proteins showing upregulated expression and downregulated expression upon HIV infection are indicated with ↑ and ↓, respectively. The image was modified from [59]. Vpu, viral protein U; SNAT1, sodium coupled neutral amino acid transporter 1; CD, cluster of differentiation; PIC, pre-integration complex; TSG101, tumor susceptibility gene 101 protein; HIV, human immunodeficiency virus; Gag, group-specific antigen; STAT3, signal transducer and activator of transcription 3; ZAP70, zeta-chain-associated protein kinase 70; CK2α, casein kinase 2α; RT, reverse-transcription.

HIV enters host cells through interacting with the proteins in plasma membrane, replicates in nucleus through integrating to host DNA, and transfers from cell to cell through exosome or cell fusion [58]. Therefore, lots of host cells and factors are involved during HIV life cycles. Some proteins related to HIV infection have been identified by subcellular proteomic studies (Figure 2). For example, HIV Vpu protein can downregulate expression of sodium coupled neutral amino acid transporter 1 (SNAT1) [55], and group-specific antigen (Gag) can regulate expression of tumor susceptibility gene 101 protein (TSG101) and vinculin [39], [60].

#### Plasma membrane proteins

The plasma membrane proteins have important roles in HIV entry and budding; therefore represent potential targets for anti-HIV drugs. A SILAC-based proteomic study has shown that compared to naïve T cells, expression of more than 100 plasma membrane proteins including SNAT1 and serine incorporators 3 and 5 (SERINC3/5) is down-regulated in HIV-infected T cells [55]. This study has also demonstrated that HIV interferes with immune metabolism of the infected host T cells by antagonizing SNAT1-mediated alanine transport through Vpr [55].

#### Exosomes

Exosomes are nanometer-sized vesicles in which many genes, proteins, and RNA are packed [61]. These packaged materials are important in regulating HIV pathogenesis, such as viral entry, budding, and trafficking [62], [63]. Using a SDS–polyacrylamide gel electrophoresis (PAGE)-based proteomic analysis, Kadiu et al. discover that microvesicles (averaged 300 nm in diameter) and exosomes (about 60 nm in diameter) facilitate viral infection through regulating a range of cell surface receptors, such as cluster of differentiation 14 (CD14), CD44R5, and vinculin [39]. Therefore, exosomes can regulate HIV-1 infection through the proteins packed in exosomes. On the other hand, the proteins packed in exosomes such as tetraspanins, flotillin, and TSG101 are also targeted by HIV-1 [60].

#### Nuclear proteome

HIV achieves the viral DNA integration into host DNA and replication in the host cell nucleus [64]. To discover how the host nuclear proteome is involved in HIV replication, Jarboui et al. have overexpressed *Tat*, the gene encoding HIV tyrosine aminotransferase, in the Jurkat T cells and performed a nuclear proteomic study by integrating MS and SILAC [45]. As a result, they identified 49 differentially-expressed proteins. Among these proteins, expression of STAT3, zeta-chain-associated protein kinase 70 (ZAP70), and casein kinase 2α (CK2α) is verified to be upregulated in the nucleus of *Tat*-overexpressing cells, while no change has been detected in cytoplasm or whole cell fractions for these three proteins. Pathway and network analyses further reveal that *Tat* expression specifically results in the nuclear enrichment of proteins participating in ribosomal biogenesis and protein homeostasis, such as cyclophilin B (CypB), and heat shock protein 90 (HSP90) [45]. Moreover, DeBoer et al. [65] report that 13 (*e.g.*, DEK) and 38 nuclear proteins (*e.g.*, dermcidin) are uniquely expressed in HIV-infected and uninfected C8166-45 T cells, respectively.

### AP-MS

An interaction network between HIV and human proteins has recently been defined by using AP−MS analysis [66]. This network contains 497 HIV−human protein–protein interactions (PPIs) involving 435 individual human proteins in HIV-infected HEK293 and Jurkat cells [66]. Following this study, Emig-Agius et al. [67] integrated the aforementioned PPI network with interfering RNA (RNAi) screening data. Consequently, they generate a PPI map of 40 HIV−human protein complexes associated with HIV infection, including proteins involved in transcription, translation, DNA replication and repair, as well as cytoskeletal regulation.

### Post-translational modifications

Post-translational modifications (PTMs), as generally enzymatic modification of proteins during or after protein biosynthesis, have very important functions in cell signaling including response to viral infection. When HIV-1 enters host cells, it activates host cell signaling pathways by regulating protein PTMs [33], [36], [68]. Below, we discuss four main PTMs.

#### Phosphorylation

To unravel the signaling events induced by HIV-1 entry, a SILAC-based quantitative phosphoproteomic study has been performed for human primary CD4^+^ T cells infected with HIV-1 [33]. The authors have identified 239 phosphorylation sites from 175 proteins after HIV infection. These proteins, including the serine/arginine repetitive matrix 2 (SRRM2, also known as SRm300), function in HIV receptor binding and mRNA splicing, among others. Another iTRAQ-based proteomic study has lately been reported to characterize the phosphorylation patterns in HIV-infected and uninfected brain parietal cortex (with or without encephalitis). In this study, they identified 112 phosphorylated proteins and 17 novel phosphorylation sites [34]. Higher phosphorylation levels in neurofilament medium polypeptide (NEFM) and myelin basic protein (MBP), *etc.* have been detected in HIV infection [34].

#### Glycosylation

Glycosylation, as a main PTM, has essential roles in HIV infection and vaccine development [69]. The glycoproteins secreted from HIV-infected T cells can help us understand the interaction between HIV and host. A glycoproteomic study focusing on N-glycosylated peptides was conducted on plasma samples from HIV patients and found that 59 human glycoproteins were related to HIV infection, including galectin-3-binding protein, L-selectin, and neogenin [70].

#### Acetylation

Acetylation of host proteins is essential for HIV-1 assembly and budding in the lipid raft microdomains of plasma membrane. The acetylation process can be mediated by HIV proteins, such as Gag [71]. Colquhoun et al. [22] obtained 103 and 174 acylated proteins using myristic acid azide and palmitic azide (which are incorporated in a manner analogous to natural acyl-Co-A) enrichment methods, respectively, of which 27 and 45 proteins were found to be expressed differentially in HIV-1 infected *vs.* uninfected CEMx174 cells.

## Recent findings in the proteomic study of HIV-associated disorders

**Figure 3:**
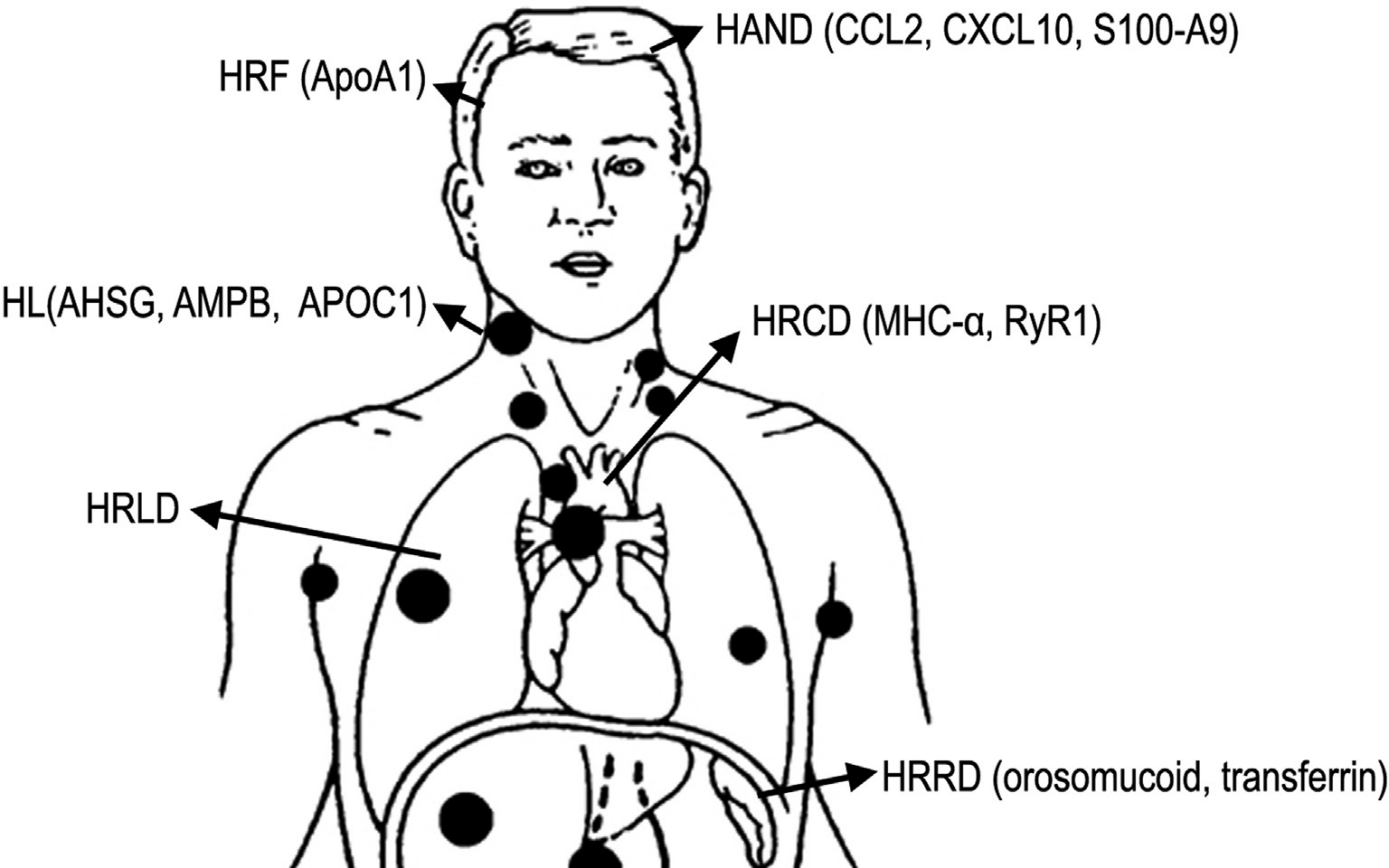
**The body map showing different HIV-associated disorders and the reported proteins** HRF, HIV-related fatigue; HL, Hodgkin’s lymphoma; HRLD, HIV-related lung disorder; HAND, HIV-associated neurocognitive disorder; HRCD, HIV-related cardiovascular disorder; HRRD, HIV-related renal disease; ApoA1, apolipoprotein A-1; AHSG, alpha-2-HS-glycoprotein; AMPB, aminopeptidase B; APOC1, apolipoprotein C-1; CCL2, C–C motif chemokine 2; CXCL10, C-X-C motif chemokine 10; S100-A9, S100 calcium binding protein A9; MHC, myosin heavy-chain; RyR1, ryanodine receptor 1.

Due to its high throughput and sensitivity, proteomics has become a useful tool in identifying biomarkers and drug targets of HIV-related diseases (Figure 3 and Table 2).

### HIV-associated neurocognitive disorders

HIV-associated neurocognitive disorders (HAND) are common in HIV-infected patients because HIV can enter the central nervous system (CNS) and cause systemic damage, such as protein expression changes. These differently-expressed proteins in cerebrospinal fluid (CSF) after HIV infection might be potential biomarkers for the diagnosis and treatment of HAND [76]. As reviewed by Price et al. [19], targeted, hypothesis-driven, and non-targeted exploratory discovery methods and proteomic technologies have been used to discover HAND-related proteins. Chemokine (C–C motif) ligand 2 (CCL2), monocyte chemoattractant protein 1 (MCP-1), C-X-C motif chemokine 10 (CXCL10), interferon gamma-induced protein 10 (IP-10), t-tau, and other proteins have been reported to be related to HIV infection [77]. In another study, the Ig κV-III chain was found to consistently respond to three HAND diagnosis biomarkers that are related to HIV neural disease severity, including HIV RNA, immune activation (neopterin), and axonal injury [54]. Similarly, by using iTRAQ-based proteomics, protein S100-A9 and metalloproteinase-9 (MMP9) are found to be associated with inflammation and cognitive impairment induced by HIV. Thus, these proteins could be potential targets for HAND treatment [72].

### HIV-related cancers

The rate of HIV-related cancers (HRCs), such as Hodgkin’s lymphoma (HL), has increased in recent years, due to the prolonged lifespan of HIV patients with the implementation of antiretroviral therapies [78]. Varnum et al. performed a proteomic analysis in plasma samples from HIV-infected patients with (*n* = 22) and without HL (*n* = 14). They discovered 57 novel candidate biomarkers related to HL, such as alpha-2-HS glycoprotein (AHSG), aminopeptidase B (AMPB), and apolipoprotein C-1 (APOC1) [75]. These proteins could be potential biomarkers for early detection of HL.

### HIV-related cardiovascular disorders

When HIV infects host cells, HIV proteins can cause cardiac dysfunction, such as cardiac stress and arrhythmia by working together with host HIV-regulated proteins [79]. For example, increased level of sCD4 is associated with rapid progression of carotid athereosclerosis [79]. An increasing number of HIV-infected individuals are reported nowadays to develop HIV-related cardiovascular disorders (HRCDs) despite a significant reduction in the viral load after antiretroviral therapy. People living with HIV infection are 50% −100% more likely to develop HRCDs than people without HIV infection [80], [81], [82]. In HIV-infected cell lines, 12 proteins have been found to be over-expressed in the plasma membrane or endoplasmic reticulum membrane, including myosin heavy chain cardiac muscle αisoform (MHC-α) and ryanodine receptor-1 (RyR1) [74]. These proteins may be used for early diagnosis or as therapeutic targets for heart disease in HIV-infected individuals.

### HIV-related fatigue

HIV-related fatigue (HRF) is a general symptom in HIV-infected patients, being reported in about 55%–65% of patients even after initiation of antiretroviral therapy [83], [84]. One proteomic study performed in clinical plasma demonstrated that apolipoprotein B (ApoB) has a negative relationship with fatigue severity in highly active antiretroviral therapy (HAART)-treated patients, whereas ApoA1 is positively related to fatigue severity in naive HIV-infected patients [37].

### HIV-related lung disorders

HIV is difficult to remove when infection occurs in the lung [85]. Thus, individuals infected with HIV are prone to develop chronic HIV-related lung disorders (HRLDs) [35]. Bronchoalveolar lavage fluid (BALF), which is directly connected to lung lesions, is helpful for discovering the pathogenesis of HIV-related pulmonary diseases. Using shotgun proteomic analysis combining with principal component analysis, Nguyen EV [86] profiled the proteome in BALF samples from HIV patients, and revealed 87 differentially-expressed proteins (such as Afamin and alpha-1-acid glycoprotein 1) compared to that of healthy control subjects [86].

### HIV-related renal disease

HIV-1 infected children are at high risk for developing HIV-related renal diseases (HRRDs), which are found in 41% of patients on initial presentation [87]. Proteomics can also provide new clues regarding early detection of HIV-related renal diseases, and target proteins for treatment. Perazzo et al. [73] found that orosomucoid, transferrin, and fibroblast growth factor-2 (FGF-2) are specific biomarkers for HIV-associated renal diseases in children.

## Mechanisms of HIV infection

### HIV–host interaction

**Figure 4:**
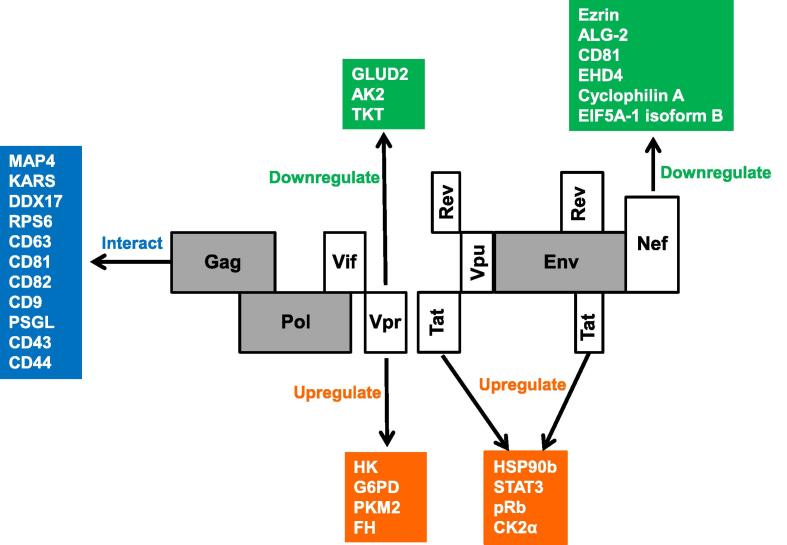
**Virus-host interactions summarized in this work** The red, green and blue squares indicate the up-regulated, down-regulated, and interactive proteins of host, respectively. MAP4, microtubule-associated protein 4; KARS, lysyl-tRNA synthetase; DDX17, DEAD-box helicase 17; RPS6, ribosomal protein S6; PSGL, P-selectin glycoprotein ligand 1; Gag, group-specific antigen; Pol, DNA polymerase; Vif, virion infectivity factor; Vpr, viral protein regulatory; Vpu, virus protein U; Rev, regulator of expression of virion proteins; Env, envelope glycoprotein gp160; Tat, tyrosine aminotransferase; Nef, neferine; HSP90b, heat shock protein 90-beta; STAT3, signal transducer and activator of transcription 3; pRb, retinoblastoma-associated protein; CK2α, casein kinase II subunit alpha; ALG-2, apoptosis-linked gene-2; EHD4, EH domain-containing protein 4;; EIF5A-1, eukaryotic translation initiation factor 5A-1; GLUD2, glutamate dehydrogenase 2; AK2, adenylate kinase 2; HK, hexokinase; G6PD, glucose-6-phosphate dehydrogenase; PKM2, pyruvate kinase M2; FH, fumarate hydratase; TKT, transketolase.

HIV-1 contains 9 genes which are either structural (*Env*, *Gag*, and *Pol*), regulatory (*Tat* and *Rev*), or accessory (*Nef*, *Vif*, *Vpr*, and *Vpu*) [7]. HIV depends mainly on host factors to complete its life cycle [58]. Identification of the host factors employed by HIV is very useful for understanding the viral invasion and host defense strategies. As summarized by Luo et al. [32], many proteomic methods have been used to identify HIV**–**host protein interactions, including AP-MS and quantitative proteomic approaches. Using these methods, the proteins, such as ubiquitin, homeodomain-containing transcription factors, and myeloid ectopic integration site (MEIS) have been discovered to interact with or be regulated by HIV [32]. Moreover, in recent years, many proteomic studies have been performed to identify interacting host**−**HIV proteins, such as Gag interacting with microtubule-associated protein 4 (MAP4) and lysyl-tRNA synthetase (KARS) (summarized in Figure 4).

### HIV Gag

In CD4^+^ T cells, HIV-1 buds from plasma membrane through its Gag protein targeting the host proteins in plasma membrane [88]. Using a proteomic approach, 22 host kinases have been identified to interact with HIV-1 Gag [21]. Gag p6 can be phosphorylated by atypical protein kinase C (aPKC) to regulate the incorporation of viral protein regulatory (Vpr) to HIV-1 virions [21]. An AP-MS-based study identified cytoskeletal network proteins (*e.g.*, MAP4) and tRNA synthetases (*e.g.*, KARS) interact with Gag [20]. Additional studies have also reported the identification and verification of DEAD-box helicase 17 (DDX17) and ribosomal protein S6 (RPS6) for their interaction with Gag [89]. Interestingly, Y-box binding protein 1 (YBX1) has been detected through affinity enrichment combined with sequential window acquisition of all theoretical fragment ions (SWATH)-MS technology and verified by immunoblotting to be a candidate protein interacting with matrix protein, a cleavage product of Gag precursor, in HIV-1 infection [19]. As reviewed by Mariani et al. [90], CD4^+^ T cell or macrophages expressing CD63, CD81, CD82, CD9, P-selectin glycoprotein ligand 1 (PSGL), CD43, or CD44 can all interact with Gag [90].

### HIV Nef

HIV-1 Nef is a main pathogenic protein of HIV and can accelerate the development of AIDS [91]. Nef promotes nanotube formation and possibly microvesicle secretion as well [45]. To identify Nef-regulated host proteins, Bregnard et al. have performed a proteomic study based on difference-GE and iTRAQ for comparative analysis between the proteomes of wild-type and *Nef*-deleted viruses. They find that compared to wild-type, Ezrin, apoptosis-linked gene-2 (ALG-2), CD81, and EH-domain containing 4 (EHD4) are enriched in *Nef*-deleted virions [53]. Analysis of the exosomes from U937 cells infected by HIV-1 overexpressing *Nef* revealed that expression of 47 microRNAs (microRNAs) was affected. These miRNAs targeted several genes for inflammatory cytokines and other pathways that are involved in HIV pathogenesis [92]. Using 2DE-MS technology, Saxena et al. [41] report that *Nef* downregulates the expression of 6 proteins in cells overexpressing HIV-1 *Nef*, such as cyclophilin A and eukaryotic translation initiation factor 5A-1 (EIF5A-1) isoform B [41].

### HIV Tat

Tat is an important regulatory protein functioning in HIV-1 replication [93]. A subcellular proteomic study integrating MS and SILAC reports that upon *Tat* expression, levels of 49 proteins, including HSP90b, STAT3, retinoblastoma protein (pRb), and CK2α, are altered in the nucleolus of Jurkat T cells. Bioinformatic analysis has revealed that Tat mainly regulates nucleolar enrichment of proteins that are involved in ribosomal biogenesis and protein homeostasis [45].

### HIV-1 Vpr

Vpr is essential for macrophage infection by HIV-1 [94]. Barrero et al. performed a SILAC analysis to characterize the Vpr-responsive proteins in macrophages. They demonstrated an increase in expression of glycolytic and citrate pathway enzymes, such as hexokinase (HK), glucose-6-phosphate dehydrogenase (G6PD), pyruvate kinase M2 (PKM2), and fumarate hydratase (FH). On the other hand, reduced levels of key mitochondrial enzymes including glutamate dehydrogenase 2 (GLUD2), adenylate kinase 2 (AK2), and transketolase (TKT) were also observed [46].

### HIV regulation of host T cells

CD4^+^ T cells are main targets for HIV infection. As reported by Navare et al. [48], 266, 60, and 22 differentially-expressed proteins (*P* ≤ 0.05) have been detected in CD4^+^ T cells 4 h, 8 h, and 20 h post HIV infection, respectively. The alteration in expression of these proteins occurs long before *de novo* production of viral proteins. The most significantly affected cellular functions according to GO analysis were reportedly protein synthesis (early upregulation) using DAVID software analysis, followed by downregulation of proteins involved in cell proliferation, DNA repair and recombination, as well as maintenance of T cell immune function [48].

### HIV regulation of host macrophages

Macrophages, other than CD4^+^ T cells, are another type of cells targeted by HIV. To identify specific proteins in monocyte-derived macrophages that are infected by HIV, Li et al. have performed SWATH-MS in conjunction with bioinformatics analyses and identified differentially-expressed proteins, such as U12-type spliceosomal complex and catalytic step 2 spliceosome. Among them, expression of 420 proteins is significantly changed after HIV-1 infection, especially nucleic acid-binding and regulatory proteins [19].

### HIV resistance mechanisms

HIV-exposed seronegative (HESN) individuals likely possess an inherent HIV resistance mechanism [95]. Identification of endogenous factors related to HIV resistance may be helpful in the development of new microbicides and treatments. Many studies have focused on HIV resistance in HESN women. Stein et al. has reported a large-scale study involving HIV-infected sex workers including 102 HESN women (HESN group) and 100 high-risk HIV-susceptible female sex workers (control group) subjects [96]. Comparative proteomic analysis reveals that myxovirus resistance protein 2 (MX2) is significantly overexpressed in HESN women. Further experimentation shows that MX2 expression could be regulated by using the long-acting contraceptive Depo-Provera [96]. In another study, iTRAQ-based proteomics of HESN individuals and two control groups (low-risk HESN and HIV-positives) has revealed that in the control groups, expression of serine proteinase inhibitor A5 (serpinA5) is upregulated, whereas expression of myeloblastin, a serine protease, is downregulated in the cervicovaginal fluid, suggesting a balance between serine proteases and their inhibitors in HIV resistance [50]. In addition, a label-free proteomic study has also been reported for paired salivary (*n* = 10) and rectal lavage (*n* = 10) fluid samples from healthy and HESN individuals, and detected 72 proteins with known immune functions, including mucins, cathelicidin, and serpins, which have defined roles in HIV defense [97]. Similarly, overexpression of cytochrome C, DnaJ homolog subfamily B member 1 (DNAJB1), poly(U)-specific endoribonuclease, *etc*. have also been detected in cationic protein-depleted secretions of cervicovaginal fluid and R5 tropic primary isolates of HIV subtype A [98].

### HIV reservoirs

Despite effective and highly active antiretroviral therapies, HIV cannot be completely eliminated due to the presence of viral reservoirs [99]. Thus, it is of utmost importance that proteins involved in HIV reservoirs should be discovered. As reviewed by Ciuffi et al. [100], many technologies, including transcriptomics and proteomics, have been used to identify the factors such as NF-kB, neogenin, and galectin-3-binding protein of HIV reservoirs. Moreover, MS-based proteomics has played strong positive roles in virological investigations for better understanding of the molecular mechanism contributing to HIV reservoirs [100]. Using MS-based proteomics, proteins related to HIV reservoirs have been identified, such as protein phosphatase-1 and small molecule activator of protein phosphatase-1 [101]. We also find that the expression levels of macrophage-capping protein (CAPG) and vesicular integral-membrane protein 36 (VIP36) are altered in the plasma membrane of A7 cells (unpublished data).

## Remarks

Proteomics is a promising technology for HIV infection research and could provide rich information on HIV–host interactions, and mechanisms underlying HIV pathogenesis, aiding in diagnosis of HIV-related diseases and new drug development [102]. Using proteomic technology, lots of new proteins related to HIV infection have been discovered. These include CD14, CD44R5, vinculin, S100 calcium binding protein A9 (S100A9), ApoA1, and FGF-2. Technological advances have greatly improved the throughout and sensitivity of proteomics. For instance, up to 8 samples can be analyzed per run of iTRAQ and about 5000 proteins can be quantified in one run as well (Table 1). Undoubtedly, there are still some technical and biological challenges associated with proteomic studies. For example, there must be enough sample amounts for proteomic study (at least 100 cells [103]). Efforts in improving the sensitivity are imperative to boost the discovery of the molecular mechanism used by the HIV to take over the host cell, mechanisms of host defense, and potential biomarkers for HIV-related diseases. Furthermore, it is also necessary to verify the identified proteins in large number of clinical samples before any potential translational applications.

## Competing interests

The authors have declared that no competing interests exist.

## Figures and Tables

**Table 1:** **New advances in HIV proteomic studies**

**Technique**	**Advantage**	**Disadvantage**	**Refs.**
Gel-based methods	Samples processed *ex vivo*; able to quantify isoforms/PTMs	Lower throughput and labor-intensive; limited to pair-wise comparisons	[36], [37], [38], [39], [40], [41], [42], [43], [44]

SILAC	Stable incorporation into all proteins	Limited to cultured cells or animal tissues only	[45], [46]

Stable ^18^O-labeling	Samples labeled *ex vivo*	Limited to pairwise comparisons	[47]

ICAT	Samples labeled *ex vivo*	Only peptides with cystine residues able to be labeled	

iTRAQ	Able to label up to eight samples in a single experiment; suitable for all kinds of samples	Co-selection of peptides by mass spectrometer leading to reporter ion intensity suppression	[48], [49], [50], [51], [52], [53]

Label-free quantitation	No labeling required; able to analyze individual samples	Requiring good replication for mass spectrometry and sophisticated software for data interpretation	[19], [54], [55]

MRM/SRM	Absolute targeted quantitation and high throughput	Difficult to identify consistent peptides to track for quantitation	[56]

TMT	Able to label up to ten samples in a single experiment; suitable for all kinds of samples	Co-selection of peptides by the mass spectrometer leading to reporter ion intensity suppression	[55], [57]

Microarray	High throughout; samples processed *ex vivo*	Requiring many kinds of antibodies; expensive	[27]

AP-MS	Direct study of protein–protein interaction	Unable to find different proteins unless combined with other technologies	[19], [20], [21]

*Note*: HIV, human immunodeficiency virus; SILAC, stable isotope labeling by amino acids in cell culture; ICAT, isotope-coded affinity tag; iTRAQ, isobaric tags for relative and absolute quantitation; MRM/SRM, multiple/selective reaction monitoring; TMT, isobaric mass tagging; AP-MS, affinity purification-mass spectrometry; PTM, posttranslational modification.

**Table 2:** **Potential biomarkers of HIV-related diseases**

**Protein**	**Regulation mode**	**Associated disease**	**Ref.**
S100A9	Negative	HAND	[72]
MMP9	Negative	HAND	[72]
FGF2, MMP2, NGAL, heptoglobin, hemopexin, lactoferrin	Positive	HRRD	[73]
EGF	Negative	HRRD	[73]
MHC-α, RyR1	Positive	HRCD	[74]
Ig κV-III chain	Positive	HAND	[54]
AHSG, APOF, APOC1	Positive	HRC	[75]
ApoB	Negative	HRF	[37]
ApoA1	Positive	HRF	[37]

*Note*: HIV, human immunodeficiency virus; S100A9, S100 calcium binding protein A9; MMP, matrix metalloproteinase; FGF, fibroblast growth factor; NGAL, neutrophil gelatinase-associated lipocalin; EGF, epidermal growth factor; MHCα, myosin heavy chain cardiac muscle α isoform; RyR1, ryanodine receptor 1; AHSG, alpha-2-HS-glycoprotein; APOF, apolipoprotein F; APOC1, apolipoprotein C-1; ApoA1, apolipoprotein A-1; ApoB, apolipoprotein B; HAND, HIV-associated neurocognitive disorders; HRRD, HIV-related renal disease; HRCD, HIV-related cardiovascular disorders; HRC, HIV-related cancer; HRF, HIV-related fatigue.
